# Assessment of combined efficacy of *Drakshaguduchyadi* gargling and *Khadiradi Vati* lozenges in curative-intent radiotherapy-induced xerostomia in head-and-neck cancer

**DOI:** 10.3389/fmed.2026.1726247

**Published:** 2026-02-02

**Authors:** K. Sivabalaji, B. N. Ashwini, Anoop Remesan Nair, P. L. Jisha

**Affiliations:** 1 Amrita School of Ayurveda, Amrita Vishwa Vidyapeetham University, Amritapuri, Kollam, India; 2 Amrita Institute of Medical Sciences, Kochi, India; 3 Ramavarma District Ayurveda Hopsital, Thrissur, India

**Keywords:** alternative therapy, Kavala, oncology, radiotherapy, sialometry

## Abstract

**Background:**

Xerostomia is a common adverse effect of radiotherapy for head-and-neck cancers, leading to significant impairment of oral health and overall quality of life. Evidence-based guidelines for managing radiation-induced xerostomia remain limited, and the majority of available options provide only temporary symptomatic relief. In Ayurveda, this condition is described under *mukharoga* (oral diseases).

**Objectives:**

The objective of the study was to evaluate the combined effect of *Drakshaguduchyadi Kavala* (gargling) and *Khadiradi Vati* lozenges on symptoms of radiotherapy-induced xerostomia using subjective questionnaires, sialometry, and salivary pH measurements.

**Materials and methods:**

A total of 20 patients were enrolled with clinically diagnosed radiotherapy-induced xerostomia. Subjective outcomes were assessed using a xerostomia questionnaire and analyzed using the Wilcoxon signed-rank test. Objective outcomes, including unstimulated salivary flow, stimulated salivary flow, and salivary pH, were evaluated using sialometry and analyzed using the paired *t*-test. Patients received *Khadiradi Vati lozenges* followed by *Drakshaguduchyadi Kavala (gargling)* three times daily for 30 days.

**Results:**

After 30 days of intervention, significant improvement was observed in multiple subjective symptoms, including oral dryness, chewing and swallowing difficulty, choking sensation, sticky saliva, impaired enjoyment of food, and difficulty in speech. Objective parameters—unstimulated and stimulated sialometry values and salivary pH—also exhibited statistically significant improvement.

**Conclusion:**

*Drakshaguduchyadi Kavala (gargling) and Khadiradi Vati lozenges* can be considered as a treatment option in radiotherapy-induced xerostomia.

**Clinical trial registration:**

Identifier CTRI/2019/12/022396.

## 1 Introduction

Xerostomia is a frequent and distressing consequence of radiotherapy to the head-and-neck region, affecting up to 80% of patients receiving treatment either as a primary modality or as an adjuvant following surgery (1). The term denotes the patient’s subjective experience of oral dryness, regardless of measurable salivary reduction. Saliva plays an essential role in maintaining oral health, and its diminished secretion can lead to multiple functional impairments, including dental caries, difficulty in chewing and swallowing, altered taste, and challenges with speech. These problems may ultimately contribute to reduced dietary intake, weight loss, and deterioration of overall wellbeing. Although several therapeutic options, such as topical agents, systemic sialogogues, and emerging devices, have been described for managing xerostomia, uniform evidence-based guidelines remain limited (2). Supportive measures such as salivary substitutes offer only temporary relief (3). In *Ayurveda,* xerostomia can be considered a *Vata-Pitta* predominant condition. *Drakshaguduchyadi kashaya* formulation is a specific preparation for *kavala* (gargling) in disorders of the oral cavity. It can pacify *tridosha*, primarily *pitta dosha*. *Khadiradi Vati* lozenges are also indicated in *mukharoga* (oral diseases), which comprise mainly *Khadira (Acacia catechu)* and *Arimeda (Acacia leucophloea).* Its properties include *tikta kashaya rasa*, *laghu ruksha guna*, and *Pittakapha pradhana tridoshaghna.* The combined nourishing effect of *Drakshaguduchyadi yoga* and the salivary-stimulatory action of *Khadiradi Vati* lozenges and the *kavala* (gargling) procedure may together alleviate the oral signs and symptoms associated with radiation-induced xerostomia (4).

## 2 Materials and methods

The study was carried out in the outpatient department of Radiation Oncology at a tertiary cancer center between 07 December 2020 and 30 April 2021. Patients aged 18–80 years, of either sex, attending follow-up after receiving curative-intent radiotherapy comprising at least 30 fractions to the head-and-neck region were screened for symptoms of xerostomia. Individuals with Sjögren’s syndrome, diabetes mellitus, or other oral conditions such as oral ulcers and mucositis were excluded from the study. Patients who were clinically diagnosed with xerostomia and had completed radiotherapy at least 1 year earlier were included irrespective of socioeconomic status. Xerostomia was objectively confirmed by an unstimulated salivary flow rate ≤ 0.3 mL/min and a stimulated salivary flow rate < 1.0 mL/min. Subjective outcomes were assessed using a xerostomia questionnaire consisting of 13 items, with each question graded on a four-point severity scale (1–4). Objective measurements included sialometry and salivary pH estimation, recorded on Days 1 and 31. Differences in objective parameters before and after treatment were analyzed using the paired *t*-test, and subjective parameters were evaluated using the Wilcoxon signed-rank test.

### 2.1 Study design

The study design was a prospective, open-label, interventional, single-arm clinical trial. The study protocol was reviewed and approved by the Institutional Ethics Committee. Written informed consent was obtained from all enrolled individuals in accordance with the Declaration of Helsinki. The clinical trial was registered.

### 2.2 Study setting and participants

A total of 24 patients were recruited for the study, of whom 20 completed all intervention sessions and outcome assessments. The study was conducted during the COVID-19 pandemic, which imposed practical constraints on recruitment and follow-up, particularly because post-radiotherapy head-and-neck cancer patients are immunocompromised and at increased risk of infection. The requirement for saliva-based objective assessments (sialometry and salivary pH estimation), which necessitated close-contact clinical procedures, further limited participant retention.

### 2.3 Outcome measures

#### 2.3.1 Subjective parameters

All the selected participants were observed at baseline (Day 1) and after treatment (Day 31). These consultations involved the assessment of the xerostomia questionnaire (Table 1). There are 13 questions, with each question graded on a four-point severity scale (1–4).

**Table 1:** Shows the questionnaire on xerostomia (17).

No:	Questions	Duration	Present/Absent	Grade
1.	Do you have pain in your mouth?			
2.	Do you have any dryness in your mouth?			
3.	Do you have trouble in eating?			
4.	Do you have problem in swallowing liquids?			
5.	Do you have problem in chewing solid foods?			
6.	Do you have problem in swallowing solid foods?			
7.	Have you choked when swallowing?			
8.	Do you have sticky saliva?			
9.	Do you have problem with sense of taste?			
10.	Do you have problem with sense of smell?			
11.	Do you have trouble in enjoying food?			
12.	Do you have cough?			
13.	Do you have trouble in talking?			

#### 2.3.2 Objective parameters

##### 2.3.2.1 Sialometry

Sialometry was used to quantify salivary flow rates. All measurements were performed at least 1 h after food intake (5).

**Figure 1:**
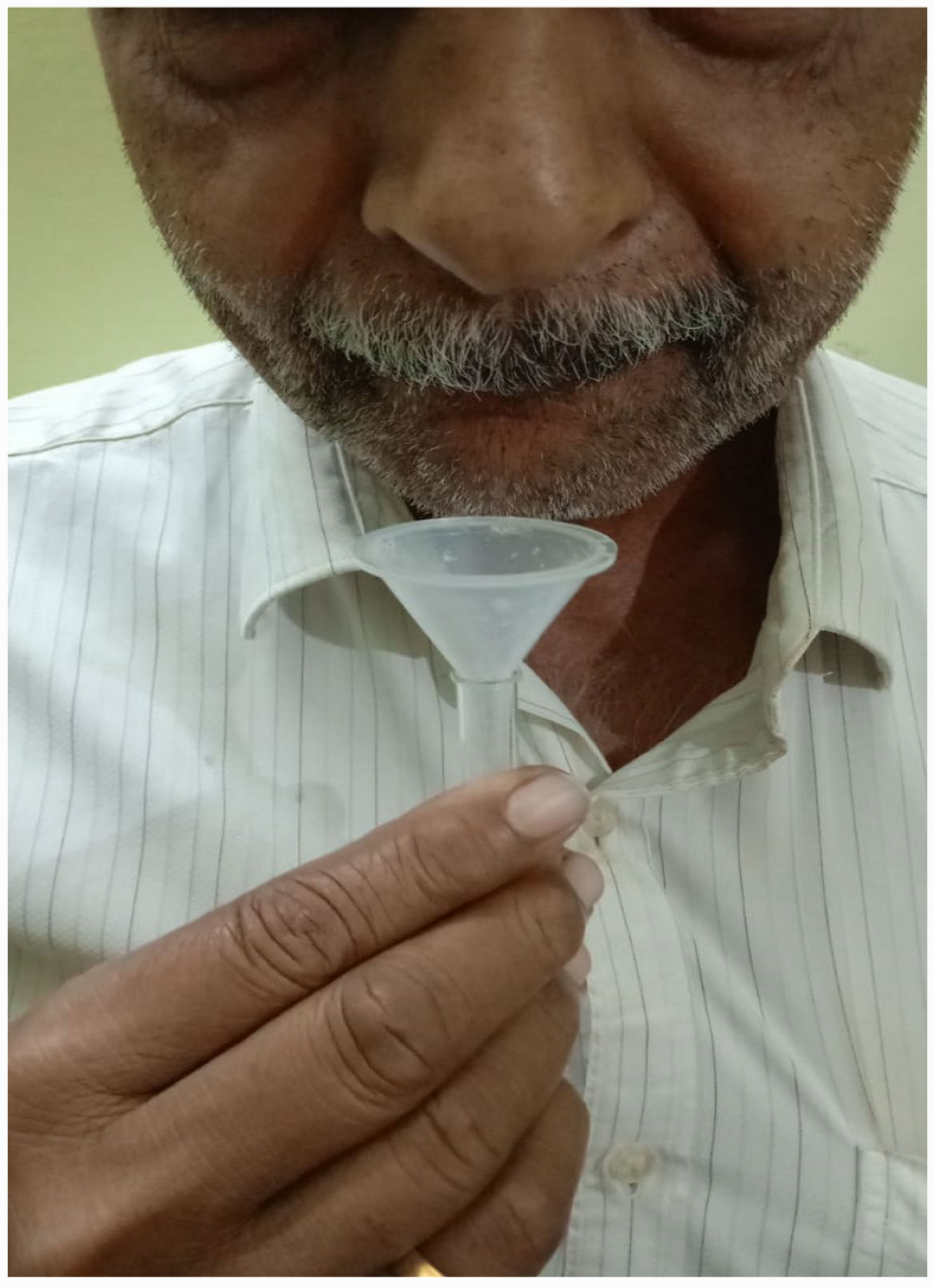
Collection of saliva.

- Unstimulated saliva collection: Participants were seated upright, with the head slightly inclined forward to allow saliva to pool naturally on the floor of the mouth. Saliva was allowed to drip passively into a measuring tube or calibrated container.
- Stimulated saliva collection: A 10% citric acid solution was applied to the tongue to provoke salivation. The patient expectorated into a measuring tube at short intervals throughout the collection period. Foam generated during the procedure was excluded from the final volume measurement (Table 2; Figure 1).

**Table 2:** Shows the grading of unstimulated and stimulated sialometry (5).

Grade	Unstimulated salivary flow measurement (mL/min)	Stimulated salivary flow measurement (mL/min)
0	0.3–0.4	>1 mL
1	0.1–0.3	0.7–1
2	<0.1	<0.7

##### 2.3.2.2 pH of saliva

Salivary pH was evaluated using pH measuring strips (Table 3).

**Table 3:** Shows the grading of pH of saliva (5).

pH of saliva	
Grade	pH value
0	<7
1	7
2	>7

### 2.4 Therapeutic intervention

Patients were administered *Khadiradi Vati for mukhadharana* (oral retention until dissolution) and *Drakshaguduchyadi kashaya for Kavala (gargling)*. The ingredients of Drakshaguduchyadi kashaya are Draksha (*Vitis vinifera*), Guduchi (Tinospora cordifolia), Sumana (*Jasminum grandiflorum*), and Darvi (Berberis aristata), and Khadiradi Vati contains Khadira Sara (*Acacia catechu*) and Arimeda twak (bark of Acacia leucophloea). A 1-g tablet of *Khadiradi Vati* was administered orally, and participants were instructed to allow slow dissolution in the oral cavity and then swallow the remaining fragments. For the Drakshaguduchyadi kashaya *kavala* procedure, 50 mL of kashaya mixed with 5 mL of *Madhu* (honey) was administered, and participants were instructed to gargle three times daily for 30 days, 1 h before food. *Mukhadharaṇa* (oral retention until dissolution) was followed by the *kavala (gargling)* procedure three times daily for 30 days. The patient’s subjective parameters, sialometry measurements, and salivary pH were assessed before and after the treatment.

### 2.5 Statistical analysis

Subjective parameters were analyzed using the Wilcoxon signed-rank test, and objective parameters, including unstimulated salivary flow, stimulated salivary flow, and salivary pH, were analyzed using the paired *t*-test. 95% confidence intervals have been included to improve precision and interpretability of the results. Statistical analyses were performed using SPSS version 19.0.

## 3 Results

**Figure 2:**
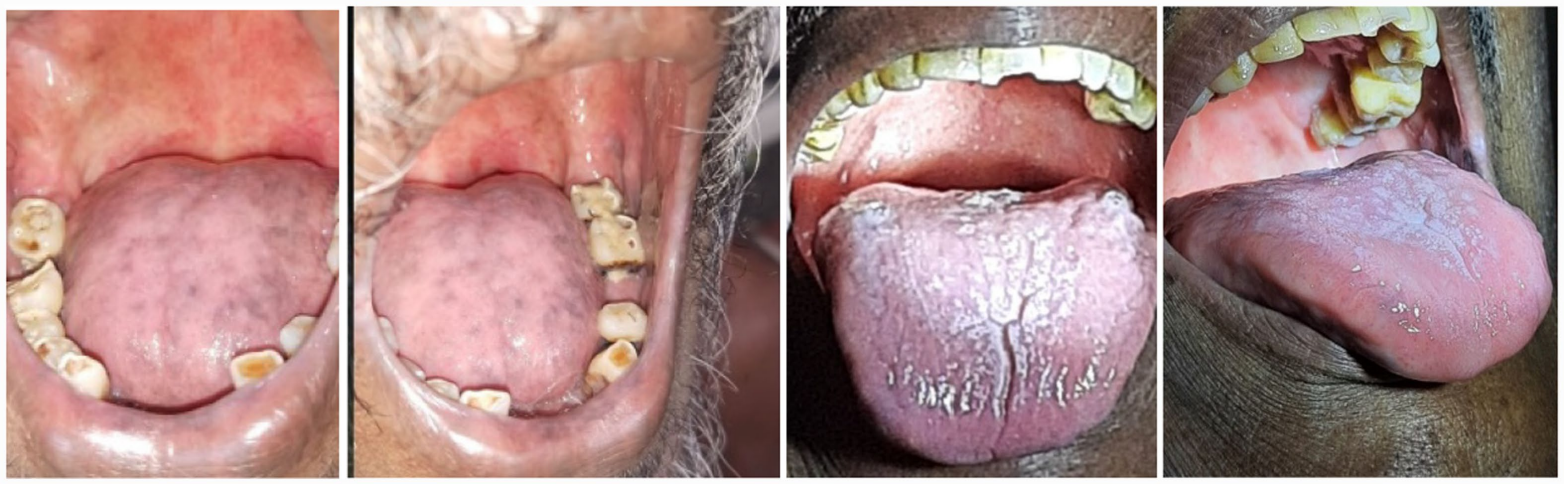
Before and after treatment.

A total of 24 patients were registered, of whom 20 completed the study. Subjective evaluation using the Wilcoxon signed-rank test showed improvement in dryness in the mouth, eating difficulty, chewing difficulty, swallowing difficulty, choking, sticky saliva, difficulty in enjoying food, and talking difficulty, which was statistically significant (Table 4; Figure 2).

**Table 4:** Shows the mean ranking of subjective parameters.

Parameters	Rank	Mean rank	Sum of ranks	*Z*-value	*p*-value
Pain in mouth_AT-negative rank	4	2.50	10.00	−2.000	0.046
Pain in mouth_BT-positive rank	0	0.00		−2.000	
Ties	16				
Total	20				
Dryness in mouth_AT-negative rank	9	5.00	45.00	−3.000	0.003
Dryness in mouth_BT-positive rank	0	0.00	0.00	−3.000	
Ties	11				
Total	20				
Eating difficulty_AT-negative rank	14	7.50	105.00	−3.49	0.001
Eating difficulty_BT-positive rank	0	0.00	0.00		
Ties	6				
Total	20				
Drinking difficulty_AT-negative rank	3	2.00	6.00	−1.732	0.083
Drinking difficulty_BT-positive rank	0	0.00	0.00	−1.732	
Ties	17				
Total	20				
Chewing difficulty_AT-negative rank	10	5.50	55.00	−2.877	0.004
Chewing difficulty_BT-positive rank	0	0.00	0.00	−2.877	
Ties	10				
Total	20				
Swallowing difficulty_AT-negative rank	12	7.17	86.00	−2.924	0.003
Swallowing difficulty_BT-positive rank	1	5.00	5.00	−2.924	
Ties	7				
Total	20				
Choking_AT-negative rank	10	5.60	56.00	−2.17	0.029
Choking_BT-positive rank	1	10.00	10.00	8	
Ties	9				
Total	20				
Sticky saliva_AT-negative rank	8	4.50	36.00	−2.64	0.008
Sticky saliva_BT-positive rank	0	0.00	0.00	0	
Ties	12				
Total	20				
Taste sensation_AT-negative rank	11	6.59	72.50	−1.00	3.317
Taste sensation_BT-positive rank	1	5.50	5.50	0	
Ties	8				
Total	20				
Smell sensation_AT-negative rank	0	0.00	0.00	−1.00	0.317
Smell sensation_BT-positive rank	1	1.00	1.00	0	
Ties	19				
Total	20				
Difficulty in enjoying food_AT-negative rank	13	7.00	91.00	−3.50	*p* < 0.001
Difficulty in enjoying food_BT-positive rank	0	0.00	0.00	0	
Ties	7				
Total	20				
Cough_AT-negative rank	7	4.00	28.00	−1.50	0.132
Cough_BT-positive rank	1	8.00	8.00	8	
Ties	12				
Total	20				
Talking difficulty_AT-negative rank	10	6.15	61.50	−2.65	0.008
Talking difficulty_BT-positive rank	1	4.50	4.50	2	
Ties	9				
Total	20				

### 3.1 Results on objective parameters

A paired *t*-test was performed to evaluate the significant differences in the mean value of sialometry and pH of saliva (Table 5). It was observed that there is a significant difference in the mean values of unstimulated salivary flow, stimulated salivary flow, and pH of saliva. The results were statistically significant.

**Table 5:** Shows the ranking of sialometry and saliva pH.

Parameters	BT mean score	AT mean score	Mean	SD	SE	*t*-value	df	Sig. (2-tailed)
Unstimulated saliva_BT Unstimulated saliva_AT	0.1265	0.24	−0.114	0.172	0.038	−2.949	19	0.008
Stimulated saliva_BT Stimulated saliva_AT	0.575	0.85	−0.275	0.172	0.194	−6.329	19	*p* < 0.001
Saliva pH_BT Saliva pH_AT	6.50	6.650	−0.150	0.286	0.064	−2.349	19	0.030

## 4 Discussion

Xerostomia is a distressing adverse effect that significantly reduces the quality of life in patients undergoing radiotherapy for head-and-neck cancer, often leading to long-term oral complications such as mucosal dryness, taste alteration, dysphagia, and increased susceptibility to infection. Radiation-induced damage results in salivary gland dysfunction mediated by oxidative stress, inflammation, and epithelial injury (6). *Drakshaguduchyadi gargling* and *Khadiradi Vati* lozenges were selected to address radiation-induced xerostomia by promoting local salivary gland stimulation, reducing mucosal inflammation, enhancing epithelial hydration, and improving oral sensory feedback, thereby restoring both salivary quantity and quality. *Drakshaguduchyadi yoga* is specifically mentioned for disorders of the oral cavity and contains ingredients with antioxidant, anti-inflammatory, and mucosal-protective properties. *Khadiradi Vati*, composed mainly of *Acacia catechu* and *Acacia leucophloea*, is also indicated for oral health and exhibits antimicrobial, anti-inflammatory, and analgesic effects. Together, these formulations may help alleviate the oral symptoms associated with radiation-induced xerostomia by supporting salivary gland function and enhancing oral mucosal hydration and integrity.

*Draksha* (*Vitis vinifera*) contains bioactive compounds with hydrating, mucosal-soothing, antioxidant, and taste-enhancing effects (7). Recent studies report that grape extracts support mucosal nourishment, improve gustatory function, and help reduce symptoms such as burning sensation and dryness. These properties may contribute to oral mucosal rejuvenation and improved taste perception (8). *Guduchi* (*Tinospora cordifolia*) exhibits anti-inflammatory, immunomodulatory, and cytoprotective actions, which support cellular repair and overall mucosal health (9). *Sumana* (*Jasminum grandiflorum*) possesses antimicrobial, soothing, and aroma-enhancing properties that may improve taste perception, increase appetite, and reduce halitosis. *Yavasa (Alhagi maurorum)* exhibits hydrating activity, supporting oral moisture. *Honey (Madhu),* with its demulcent, antimicrobial, and soothing properties, may promote salivary secretion and enhance oral lubrication through its natural viscosity and bioactive components (10).

The act of *Kavala* (*gargling*) exerts increased mechanical pressure inside the oral cavity, which helps strengthen the muscles of the cheeks, tongue, lips, and soft palate, improving movement during chewing and pronunciation. *Gargling* may help regulate and maintain the pH of the oral cavity (11).

*Khadiradi Yoga* contains predominantly bitter and astringent phytoconstituents with cleansing and mucosa-clarifying actions. These properties help reduce oral coating, improve local mucosal clearance, and alleviate the feeling of sticky saliva. The formulation’s cleansing and antimicrobial effects may also help prevent secondary oral complications. *Khadira (Acacia catechu),* the principal ingredient, contains catechuic acid, protocatechuic acid, phloroglucinol, flavonoids, and catechin compounds known for their antioxidant, anti-inflammatory, antimicrobial, and mucosal-soothing effects (12). *Arimeda (Acacia leucophloea)* has analgesic and anti-inflammatory activity, contributing to relief from oral discomfort. *Syzygium aromaticum (Lavanga)* provides local analgesic and wound-healing properties due to its major active component, eugenol, which also offers antimicrobial action and reduces dental and mucosal pain. *Glycyrrhiza glabra (Yashtimadhu)* contains glycyrrhizin and its derivative carbenoxolone, which has been shown to enhance mucus production, support mucosal protection, and reduce inflammation, thereby promoting oral comfort and improved lubrication (13).

*Laksha* (*Kerria lacca*) supports oral tissue integrity and contributes to the physiological restoration of salivary gland function. Its rejuvenating properties help maintain oral mucosal health and may aid in reducing discomfort. *Woodfordia fruticosa (Dhathaki), Plectranthus vettiveroides (Ambu), Chrysopogon zizanioides (Sevya),* and *Cinnamomum verum (Twak)* possess hydrating and mucosal-moisturizing actions that support oral lubrication. *Gmelina arborea (Katphala)* and *Berberis aristata (Darvi)* are traditionally indicated for oral cavity disorders and exhibit taste-enhancing *(ruchya)* and analgesic *(vedanāsthāpaka)* effects, which may contribute to improved oral comfort. *Emblica officinalis (Amalaki)* is a rich source of vitamin C and hydrolysable tannins, providing strong antioxidant effects. Gallic acid, a major phenolic constituent of *Triphala*, has exhibited potent antioxidant activity and the ability to suppress cancer cell growth (14). *Acorus calamus (Vacha)* has specific action in the throat and oral cavity; it promotes oropharyngeal mucosal wellbeing, improves vocal clarity, and stimulates appetite and taste sensation. *Myristica fragrans (Jatiphala)* enhances gustatory function, reduces oral malodor (anti-halitosis effect), alleviates dryness, and improves taste perception. *Prunus cerasoides (Padmaka)* and *Alhagi maurorum (Yavasa)* help alleviate dryness and support oral hydration. Camphor (*Karpura*) has beneficial effects in reducing oral dryness and improving altered taste perception (15).

*Gargling* allows direct contact of the formulation with the oral mucosa and salivary duct openings. *Drakshaguduchyadi* formulation contains agents with mucoprotective, antioxidant, and anti-inflammatory properties, which may reduce radiation-induced mucosal inflammation and improve epithelial hydration. The cooling and unctuous nature of the formulation may also enhance parasympathetic salivary reflexes, contributing to increased unstimulated salivary flow. *Khadiradi Vati lozenges* provide prolonged mechanical and gustatory stimulation within the oral cavity, which is known to activate salivary reflex pathways. Additionally, sustained local exposure to phytoconstituents with astringent, antimicrobial, and wound-healing properties may improve mucosal integrity and oral sensory function, thereby enhancing both stimulated salivary secretion and subjective comfort. The combined use of gargling and lozenges addresses xerostomia through complementary mechanisms; gargling primarily improves mucosal hydration and reduces inflammation, while lozenges maintain prolonged salivary stimulation and sensory feedback. This multimodal approach may explain the significant improvements observed in both objective and subjective parameters.

None of the participants in the present study reported allergic or adverse reactions to *Drakshaguduchyadi yoga* or *Khadiradi Vati*. In the current study, patients were advised to report any symptoms suggestive of allergy, such as oral burning, swelling, itching, rashes, or breathing difficulty; no such events occurred during the treatment period (16).

### 4.1 Limitations

As an open-label, single-arm study without blinding or a comparator group, the design inherently limits the strength and generalizability of the conclusions. Although 24 patients were initially enrolled, only 20 completed the study due to COVID-19-related challenges, reduced feasibility of repeated in-person visits, and the heightened vulnerability of immunocompromised individuals. The absence of a control arm, limited external validity, and the relatively short 30-day follow-up period further constrain interpretation of the outcomes. Future randomized controlled trials with larger sample sizes and extended follow-up durations are recommended to provide more robust evidence.

## 5 Conclusion

The findings of this study indicate that *Drakshaguduchyadi Kavala* and *Khadiradi Vati lozenges* may improve symptoms of radiotherapy-induced xerostomia. No serious adverse effects were reported, and treatment compliance was good. Randomized controlled studies with larger sample sizes need to be done to generate stronger evidence.

## Data Availability

The original contributions presented in the study are included in the article/supplementary material, further inquiries can be directed to the corresponding author.
